# Linkage between Increased Nociception and Olfaction via a *SCN9A* Haplotype

**DOI:** 10.1371/journal.pone.0068654

**Published:** 2013-07-10

**Authors:** Dirk Heimann, Jörn Lötsch, Thomas Hummel, Alexandra Doehring, Bruno G. Oertel

**Affiliations:** 1 Institute of Clinical Pharmacology, Goethe - University, Frankfurt am Main, Germany; 2 Fraunhofer Institute of Molecular Biology and Applied Ecology - Project Group Translational Medicine and Pharmacology (IME-TMP), Frankfurt am Main, Germany; 3 Smell and Taste Clinic, Department of Otorhinolaryngology, University of Dresden Medical School, Dresden, Germany; Boston Children’s Hospital and Harvard Medical School, United States of America

## Abstract

**Background and Aims:**

Mutations reducing the function of Na_v_1.7 sodium channels entail diminished pain perception and olfactory acuity, suggesting a link between nociception and olfaction at ion channel level. We hypothesized that if such link exists, it should work in both directions and gain-of-function Na_v_1.7 mutations known to be associated with increased pain perception should also increase olfactory acuity.

**Methods:**

*SCN9A* variants were assessed known to enhance pain perception and found more frequently in the average population. Specifically, carriers of *SCN9A* variants rs41268673C>A (P610T; n = 14) or rs6746030C>T (R1150W; n = 21) were compared with non-carriers (n = 40). Olfactory function was quantified by assessing odor threshold, odor discrimination and odor identification using an established olfactory test. Nociception was assessed by measuring pain thresholds to experimental nociceptive stimuli (punctate and blunt mechanical pressure, heat and electrical stimuli).

**Results:**

The number of carried alleles of the non-mutated *SCN9A* haplotype rs41268673C/rs6746030C was significantly associated with the comparatively highest olfactory threshold (0 alleles: threshold at phenylethylethanol dilution step 12 of 16 (n = 1), 1 allele: 10.6±2.6 (n = 34), 2 alleles: 9.5±2.1 (n = 40)). The same *SCN9A* haplotype determined the pain threshold to blunt pressure stimuli (0 alleles: 21.1 N/m^2^, 1 allele: 29.8±10.4 N/m^2^, 2 alleles: 33.5±10.2 N/m^2^).

**Conclusions:**

The findings established a working link between nociception and olfaction via Na_v_1.7 in the gain-of-function direction. Hence, together with the known reduced olfaction and pain in loss-of-function mutations, a bidirectional genetic functional association between nociception and olfaction exists at Na_v_1.7 level.

## Introduction

Rare variants of voltage-gated sodium channels Na_v_1.7 [1] coded by homozygous nonsense mutations in the *SCN9A* gene result in truncated, non-functional sodium channels that produce ion currents no greater than background [2]. Na_v_1.7 channels are expressed on pain receptors and afferent nociceptive neurons [3], [4]. Thus these [2] and other loss-of-function variants cause a complete [5], [6] or a partial [7] insensitivity to pain. Due to its important role in pain perception and because their expression is restricted to peripheral sensory neurons [8], Nav1.7 channels are presently reckoned as a promising target for the development of new analgesics [9], [10].

However, expression of Na_v_1.7 channels is not confined to nociceptive neurons. They are also expressed on olfactory neurons [11], where they have been shown to be the most predominant sodium channel in rodents [12]. In the absence of functional Na_v_1.7 channels, olfactory neurons continue to produce action potentials after excitation by odors but fail to initiate synaptic signaling [13]. Hence a common co-phenotype in patients carrying loss-of-function variants of *SCN9A* is anosmia [2], [5], [6], [13] which continues to raise scientific interest in pain-focused reports [14]. This suggests a link between nociception and olfaction at ion channel level [15].

Based on the physiological function of Na_v_1.7, we hypothesized that this link should work in both directions. That is, gain-of-function *SCN9A* variants, causing severe pain attacks by enhancing channel activation or impairing channel inactivation associated with inherited primary erythromelalgia or the paroxysmal pain syndrome [16]–[26], should also cause enhanced olfaction. To prove the hypothesis, human carriers of more frequent *SCN9A* variants, i.e., rs6746030C>T (R1150W) found at an allelic frequency in caucasians of ∼14%, and associated with decreased heat thresholds [27], and rs41268673C>A (P610T), found at an allelic frequency of ∼4% and also modulating nociception [28], were studied for their joint nociceptive and olfactory effects.

## Methods

### Subjects and Design

The study followed the Declaration of Helsinki and was approved by the Ethics Committee of the Goethe-University Frankfurt am Main, Germany. Informed written consent from each participating subject had been obtained. In general, data storage and handling was performed in compliance with the data protection act of the German federal state Hessen, i.e., Hessisches Datenschutzgesetz (HDSG), and the German Federal Data Protection Act, i.e., Bundesdatenschutzgesetz (BDSG). All participants were of Caucasian ethnicity by self-assignment. Three genotypic groups were enrolled, i.e. non-carriers of the minor alleles of *SCN9A* rs41268673C>A (P610T) and rs6746030C>T (R1150W), and carriers of either of the minor alleles. Power calculations based on published data [29], [30] had resulted in group sizes of 10 to observe differences of 15% in nociceptive thresholds [30] and of 16 to observe clinically relevant differences of 5.5 points [31] in an olfactory score, both at a power of 80% and an α error of 5%. To further increase the power, the group size for wild type subjects was roughly doubled to 40. The enrolled cohort finally consisted of 29 healthy men and 46 women (age 20–43 years, mean ± standard deviation 26.1±4.2 years) of whom 40 were wild type with respect to both *SCN9A* loci, 14 carried the minor rs41268673A allele and 21 the minor rs6746030T allele. The subjectś actual health was assessed by medical history, physical examination including vital signs, and routine clinical laboratory test results.

### Genotyping

Compared with the rare gain-of-function variants associated with pathologically syndromes of exacerbated pain episodes, the selected variants confer only moderate pain enhancing effects. However, their frequency is high enough to allow for the present controlled study investigating the link between enhanced nociception and olfaction at Na_v_1.7 sodium channel level. The variant rs6746030C>T causes an amino acid exchange from arginine to tryptophan at position 1150 located in the intracellular loop between transmembrane domains II and III of Na_v_1.7, a region of the channel with unknown function. Thus, the exact mechanism of the enhanced pain sensitivity is unclear. Electrophysiological assessments showed a slightly lesser voltage-dependent slow inactivation of Na_v_1.7 1150W (encoded by the minor A allele) proposed to cause changes in the setting of the threshold and firing frequency of nociceptive neurons and thus to render the neurons more sensitive to painful stimuli [27]. Furthermore, Na_v_1.7 1150W produced hyperexcitability when expressed in rat dorsal root ganglion neurons [22].The other variant, rs41268673C>A, causes an amino acid exchange at position 610 from proline to threonine, however, its molecular consequences are unknown yet.

DNA samples were available from 822 unrelated subjects of Caucasian ethnicity (mostly medical students) who had consented into genotyping. Ethics approval for genotyping had been obtained. Genomic DNA was extracted from 200 µl blood using the EZ1 DNA Blood 200 µl Kit on a BioRobot EZ1 Workstation (Qiagen, Hilden, Germany). Genotyping for the functional variants SCN9A rs41268673C>A and rs6746030C>T was done from genomic DNA by means of Pyrosequencing™ assays on a PSQ 96 MA System (Qiagen, Hilden, Germany) using the PyroMark Gold Q96 Reagents set (Qiagen, Hilden, Germany). PCR reactions were performed in a 25 µl assay volume on a Mastercycler ep gradient S instrument (Eppendorf, Hamburg, Germany), using the HotStar plus Taq Polymerase system (Qiagen, Hilden, Germany) and SNP-specific PCR primers (for rs41268673 forward primer: 5′-GCGACGCAGCAGTAACATCAG-3′ and reverse primer: 5′-biotin- TGTAAAACGTCCTTACGCTGTCA-3′; for rs6746030: forward primer: 5′-TTTGGTTGAGGGAGTATCACAGAA-3′ and reverse primer: 5′-biotin-TTGTAGCAGGTTTTCCTGATGTTC-3′, respectively). The PCR was done with an initial denaturation step for 5 min at 95°C, 45 cycles with a 30 second denaturation step at 95°C, an annealing step at primer-specific temperatures (56°C for rs41268673C>A and 58°C for rs6746030C>T) for 30 s and an elongation step at 72°C for 30 seconds, followed by a final elongation step at 72°C for 5 min. The PCR products (25 µl) were used in the Pyrosequencing analyses as previously described [32] with the sequencing primers 5′- CCAAGCCAGTAGGTCC-3′ for rs41268673C>A and 5′-CTTTCTTGTCAGGTTGTG-3′ for rs6746030C>T, respectively. For both assays, three samples of each genotype were selected, sequenced by conventional means (LGC GmbH, Berlin, Germany) and used as positive controls during Pyrosequencing™. Linkage disequilibrium (parameters D′ and r^2^ [33], [34]) between SNPs was analyzed using the Haploview software [35]. Haplotypes were obtained *in-silico* using PHASE software [36] after having established that the two SNPs were located on the same haploblock according to the solid spine of LD algorithm implemented in Haploview.

### Assessment of Nociceptive and Olfactory Sensitivity

#### Olfactory testing

The olfactory test was based on felt-tip pens that contained a solution of an odorant instead of liquid dye (“Sniffin’ Sticks”: Burghart, Wedel, Germany [37]). The pen’s cap was removed by the experimenter for approximately 3 s and the pen’s tip was placed 1–2 cm in front of the nostrils, in the case of triplet pen presentation at an interval of approximately 3 s. Three main components of olfactory function were assessed birhinally.

Specifically, **odor thresholds** were obtained for the rose-like odor phenylethylalcohol presented in 16 successive 1∶2 dilution steps starting from a 4% solution. Using a three-alternative forced-choice task (3-AFC) and a staircase paradigm starting at low phenylethylalcohol concentrations, one pen with the odorant and two blanks were presented at each dilution step. Two successive correct or one incorrect identification triggered the reversal of the staircase. The odor threshold was the mean of the last four out of seven staircase reversals (normal values >6 [29]).

**Odor discrimination** was determined with 16 triplets of pens, two containing the same odorant and the third a different, “target” one (i.e., (target/non-target) butanol/2-phenyl ethanol, isoamylacetate/anethole, anethole/eugenol, limonene/fenchone, (-)carvone/(+)carvone, eugenol/cinnamon aldehyde, dihydrorosenoxide/menthol, acetaldehyde/isoamylacetate, citronellal/linalool, pridine/limonene, limonene/citronellal, eucalyptol/dipyridyl, dipyridyl/cyclopentadecanoate, butanol/fenchone, octylacetate/cinnamon aldehyde, carvone/acetaldehyde). The discrimination performance was assessed employing a 3-AFC task (normal score ≥11 correct discriminations).

Finally, **odor identification** was determined with 16 odors (i.e., orange, leather, cinnamon, peppermint, banana, lemon, liquorice, turpentine, garlic, coffee, apple, clove, pineapple, rose, anise and fish) using a four-alternative forced-choice task with presentation of a list of four descriptors for each pen (normal score: ≥12 correct identifications).

The evaluation of olfactory performance followed the clinically established procedure consisting of calculating a composite “TDI score” (“**T**hreshold **D**iscrimination **I**dentification”) as the sum of the scores from the three subtests [38]. Pathologic olfactory function is indicated by TDI ≤30.5, with the separation of hyposmia (30.5≥ TDI >15.5) from functional anosmia at TDI ≤15.5 [29].

#### Experimental pain threshold testing

**Heat pain** thresholds, as described previously [30], were obtained using a Thermal Sensory Analyzer (Medoc Advanced Medical Systems Ltd., Ramat Yishai, Israel). Heat stimuli (range 32–52.5°C) were applied with a 3×3 cm^2^ thermode placed on a skin area of the left or right (randomized) volar forearm. Temperature was continuously increased by 0.3°C/s starting at 32°C. The subject pressed a button at the first sensation of pain, defined as the heat pain threshold, which triggered cooling of the thermode by 10°C/s. Heat stimuli were applied eight times at intervals of 25–35 s. The median of last five responses was used as heat pain threshold because in previous experiments a plateau was reached after the first three measurements.

**Cold pain** thresholds were determined with the same equipment as heat pain thresholds. Cold stimuli (range 32–0°C) were applied with the 3×3 cm^2^ thermode placed on a skin area of the volar forearm opposite to the side used for the determination of the heat pain threshold. Temperature was continuously lowered from 32°C to 0°C by 1°C/s. Since preliminary experiments had not indicated any directed tendency of the first measurements, cold pain thresholds were measured only five times and the median of these measurements was submitted to the statistical analysis.

Pain thresholds to **electrical stimuli** were obtained using a constant current device (Neurometer® CPT, Neurotron Inc., Baltimore, MD) that delivered sine-wave stimuli at 5 Hz applied via two gold electrodes placed on the medial and lateral side of the mid-phalanx (middle finger of the right hand as default-testing site). Their intensity was increased from 0 to 20 mA by 0.2 mA/s. During the test, subjects kept a button continuously pressed until they felt pain and interrupted the current by releasing the button. The electrical current at which this occurred was defined as the pain threshold. Measurements were repeated five times at intervals of 30 s and the median was analyzed.

Pain thresholds to **blunt pressure** were obtained using a pressure algometer with a circular and flat probe of 1 cm diameter (Commander Algometer, JTECH Medical, Midvale, Utah). It was placed perpendicularly onto the mid-phalanx of the right middle finger. The pressure was increased at a rate of approximately 9 N/cm^2^ per second until the subject indicated pain. The increase in pressure was controlled manually by the investigator and stopped once the subject reported pain. The maximum pressure applied was recorded automatically. The procedure was repeated five times at intervals of 30 s. Mechanical pain threshold to blunt pressure was the median of the five measurements.

### Statistics

The hypothesis of the assessments was that *SCN9A* SNPs previously associated with high pain due to sodium Na_v_1.7 hyperactivity are also associated with increased olfactory acuity. Therefore, the analysis firstly aimed at identifying the *SCN9A* variant that modulated an olfactory parameter by submitting each olfactory parameter (odor threshold, discrimination, identification) to multiple linear regression analysis (SPSS 21 for Linux, SPSS Inc., Chicago, USA) with stepwise inclusion of *SCN9A* variants. As the hypothesis was directed, i.e., higher sensitivity was the expected effect of the variants [22], post-hoc tests were one-sided using the Jonckheere–Terpstra trend test as a gene dose effect was expected. The same test was applied subsequently to identify whether the *SCN9A* variant identified as modulating olfaction also modulated pain. The α-level was set at 0.05 and corrected for multiple testing (Bonferroni) when indicated.

## Results

In the random sample of 822 subjects screened for this study, the observed minor allelic frequencies were 3.6% for variant rs41268673C>A and 14% for rs6746030C>T; Hardy-Weinberg equilibrium was preserved (χ^2^ test: p>0.05). Further analyses were done in the study cohort of 75 subjects selected for *SCN9A* genotypes. Linkage disequilibrium parameters between the two *SCN9A* SNPs were D′ = 100 and of r^2^ = 2 and both SNPs were located together in a haploblock. Therefore, association analyses focused on haplotypes. The non-mutated haplotype rs41268673C/rs6746030C was found at an allelic frequency of 75.7% (and in the random sample of n = 822 it had a frequency of 82.5%), the haplotype with major allele of the rs41268673 SNP and variant (minor) allele of the rs6746030SNP, i.e., rs41268673C/rs6746030T, was found at an allelic frequency of 13.8%, and the haplotype with variant allele of the rs41268673 SNP and major allele of the rs6746030 SNP, i.e., rs41268673A/rs6746030C, had an allelic frequency of 10.5%. A haplotype with both minor alleles was not found.

**Figure 1:**
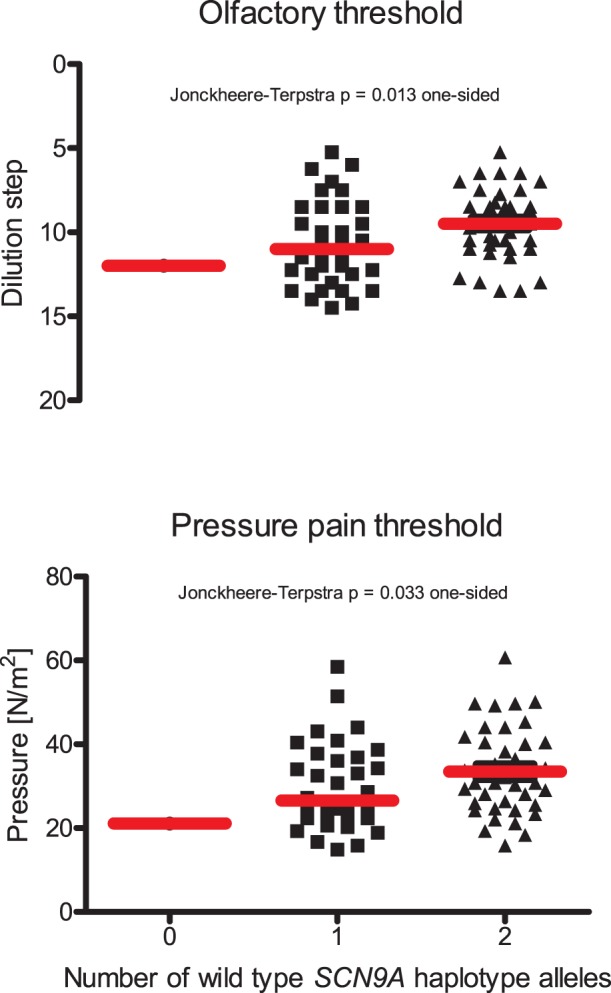
Olfactory and pressure pain thresholds increased with increasing number of wild-type *SCN9A* haplotype alleles rs41268673C/rs6746030C. Please note that the olfactory threshold to phenylethylethanol is scaled inversely as the ordinate indicates dilution steps, with higher numbers denoting lower thresholds. Thus, both sensory thresholds increase in the same direction indicating that the presence of *SCN9A* variants lowers the thresholds making the subject more sensitive against smell and pain stimuli. Single data and the median (thick horizontal red line) are shown. Significant genotype effects were also found when excluding group zero.

None of the subjects reported any perception of pain or smell sensitivities differing from the normal status. Most subjects (n = 72) were normosmic (TDI score ≥30.5 [29]). Among the three hyposmic subjects (TDI of 28, 29 and 30, respectively), the wild type haplotype rs41268673C/rs6746030C was carried in a total of five copies. Multiple linear regression analysis of olfactory parameters identified the *SCN9A* wild-type haplotype as the genetic factor that statistically significantly influenced olfactory thresholds (p = 0.024). An increasing number of non-mutated haplotype alleles was associated with a higher threshold, i.e., with the detection limit of volatile phenylethylethanol at lower dilutions (Figure 1). This was reflected in a statistically significant trend (Jonckheerés trend test p = 0.013 one-sided). The same wild-type haplotype also modulated the pressure pain threshold (Jonckheerés trend test p = 0.033 one-sided). In addition, pressure pain thresholds were significantly modulated by the rs41268673A/rs6746030C haplotype (p = 0.022) in the expected direction of carriers having lower thresholds than wild-type subjects.

The remaining olfactory parameters and pain thresholds to heat and punctate mechanical stimuli differed only insignificantly between the identified haplotypes, but still observed variations displayed a tendency in the hypothesized direction. Specifically, the composite olfactory TDI scores dwindled with each additional wild-type haplotype from a value of 40 in the carrier of zero wild-type haplotypes (n = 1), to 37±3.4 in carriers of one wild-type haplotype (n = 34), to 35.4±3.2 in carriers of two wild-type haplotypes (n = 40). Likewise, the heat and cold pain threshold tended in the direction expected from animal experiments [39], i.e., the heat pain threshold was lower while the cold pain threshold was higher in carriers of one wild-type haplotype (45.5±3.3°C and 13.1±9.6, respectively; n = 34) compared to carriers of two wild-type haplotypes (46.5±2.2°C and 9.9±9.3°C, respectively; n = 40).

## Discussion

The study hypothesis that *SCN9A* gain-of-function variants should cause increased olfactory acuity as *SCN9A* loss-of-function variants induce anosmia, was verified by inverse concluding that the presence of wild-type genetics was associated with the opposite effects, i.e., reduced pain perception and olfactory acuity. This way we verified that there is a robust link between nociceptive and olfactory acuities at Na_v_1.7 ion channel level [15] and that this link works in both directions, rather than in the already known negative direction of sensory function loss [2], [5], [6].

Although patients with hereditary channelopathy-associated insensitivity to pain due to *SCN9A* channelopathy [40] were reported to be otherwise healthy except anosmia [2], pathophysiological implications of *SCN9A* are not limited to pain and olfaction [41], [42]. Gustation may be another sense affected by functional changes in the Na_v_1.7 sodium channels. In particular, *SCN9A*, together with *SCN2A* and *SCN3A*, was found in cells expressing TRPM5, which is attributed to the initial sodium influx in sweet, bitter, and umami taste cells [43]. There it is likely to account for the tetrodotoxin-sensitive sodium currents [43]. Recently, a new *SCN9A* missense variant (G2567A leading to an amino acid exchange from glycine to aspartic acid at position 856) has been linked to abnormal limb development in three male family members reporting erythema and burning pain in their distal extremities [44]. Further associations have been shown with the hereditary generalized epilepsy with febrile seizures [45] and with other hereditary epilepsy phenotypes [45], [46]. Outside the nervous system, Na_v_1.7 plays a role in cell migration, endocytosis, and secretion during progression of aortic intimal hyperplasia [47]. *SCN9A* is also expressed in human prostate cancer [48], [49], for which it has been proposed as a biomarker [50]. Thus, there are plenty of other physiological systems and functions than pain and olfaction that are likely to be affected by functional *SCN9A* variants.

Neither are pathophysiological consequences associated with *SCN9A* restricted to nociception and olfaction, nor is the link between pain and smell perception restricted to *SCN9A*. Indeed, these two evolutionarily ancient sensory systems [51], [52] share more common features on the molecular level. For example, the cannabinoid system not only plays an important role in nociception [53], but cannabinoids like 2-arachidonoyl-glycerol are also synthesized in olfactory receptor neurons of Xenopus laevis larvae. There, they control odor detection thresholds via cannabinoid receptor activation [54]. Also transient receptor potential cation channel, subfamily V, member 1, activated by heat, protons and capsaicin are expressed at nociceptors, pain relevant brain areas [55] and also in the olfactory bulb [56].

Odor thresholds have been reported to be unrelated to higher cognitive factors, whereas proficiency in executive functioning and semantic memory contributed significantly to odor discrimination and identification performance [57]. Since only olfactory thresholds were presently increased, while discrimination and identification remained unaffected, this supports a more peripherally than centrally localized effect of increased Na_v_1.7 function. The same applies for the increased nociception. Only recently it has been shown that small nerve fiber neuropathy is caused by the expression of gain of function mutant sodium channels in small diameter peripheral axons [58]. This would be consistent with the reported predominant localization of Na_v_1.7 channels, which are preferentially expressed within dorsal root ganglion neurons and their small-diameter peripheral axons [59].

The present results establish olfactory effects as a class effect of future Na_v_1.7 modulating analgesics [9], [10]. Olfactory side effects seem unavoidable, as opposed to other side effects, such as cardiovascular side effects. The latter seem to be owed to an insufficient specificity for Na_v_1.7, e.g. benzazepinones binding at Na_v_1.5 [60] that is expressed on the cardiac muscle [61]. Although this might be a disadvantage for future analgesics, when thinking in the opposite direction, Na_v_1.7 activation may be suitable as a cure for remnant olfactory function. However, at this point, this assumption is purely speculative. This is amongst other things because the exact mechanism of hyposmia, for instance, in patients with post-viral olfactory loss or patients with sinunasal disease, is often unclear. While olfactory receptor neurons (ORN) are still present they appear to be deformed or some of their cilia may be lost in post-viral olfactory loss [62], ORNs appear to be normal in sinunasal disease and the olfactory disorder seems to originate from functional changes at cellular level [63]. Even in patients with posttraumatic olfactory loss it is uncertain whether shearing of the olfactory fiber is the major contributor to olfactory loss [64] or whether it results from actual brain damage. Moreover, the success of a Na_v_1.7 activating drug as a cure for remnant olfactory function will depend on its therapeutic range and its nociceptive side effects. Achieved olfactory effects would have to be greater than those presently observed. In fact, at least 5.5 points difference in the composite TDI score are required for a perceived change of olfactory function [31]. While systemic nociceptive side effects may be avoided by a local intranasal administration, the possibility of local painful side effects due to Na_v_1.7 activation on nociceptors still exists. Taken together, the utility of Na_v_1.7 modulating agents as a cure for olfactory disorders remains uncertain. Nevertheless, it is worth assessing as there is a need for such drugs. Olfactory loss in otherwise healthy people causes suffering, as it is associated with a reduction in the enjoyment of food [65], will impact the quality of life and may greatly affect certain professionals who rely on their sense of smell. Cures, on the other hand, are rare [66] and restricted to a few agents with local decongestive or anti-inflammatory actions such as metazolines [67] or corticosteroids [68] and further substances such as the N-methyl-D-aspartic acid antagonist caroverine [69] or the phosphodiesterase inhibitor theophylline [70].

Present results verified the hypothesis that there is a link between nociception and olfaction at Na_v_1.7 sodium channel level in the gain-of-function direction, adding to the previously known opposite effects of loss-of-function mutations in the *SCN9A* gene and hence, establishing that the link is bidirectional. A wild-type *SCN9A* haplotype composed of rs41268673C/rs6746030C alleles was identified to be linked with a comparatively reduced pain perception and olfaction acuity. This haplotype is frequent and found in a random sample of Caucasians at an allelic frequency of 82.5%. It is one of the few human genetic variants modulating olfactory function. However, as often seen with frequent genetic variants [71], it confers only a small effect size resulting in a measurable although not yet perceivable change in olfaction without pathologic enhancement of nociception. From a pharmacological point of view, the results indicate that modulation of Na_v_1.7 may serve different clinical purposes. This includes deactivation for the treatment of pain but also activation as a cure of diminished olfaction for which a medical need exists [66]. Nevertheless, pharmacological modulation of Na_v_1.7 function will always be associated with nociceptive and olfactory effects in both directions.
